# Retraction: Astakine 2—the Dark Knight Linking Melatonin to Circadian Regulation in Crustaceans

**DOI:** 10.1371/journal.pgen.1005222

**Published:** 2015-04-27

**Authors:** Apiruck Watthanasurorot, Netnapa Saelee, Amornrat Phongdara, Sittiruk Roytrakul, Pikul Jiravanichpaisal, Kenneth Söderhäll, Irene Söderhäll

The authors wish to retract this publication due to errors, brought to their attention by a reader, which were made in preparing figures. The figures in question are 2D, 6B and 6G in which the actin panels have been duplicated, Fig 5C and 5D where too much contrast or brightness was used, Fig 7C in which the second panel from the top has been scaled incorrectly and finally 3B and 8B which were assembled inaccurately.

All of these published figures were prepared and assembled prior to publication by the first author Dr. Apiruck Watthansurorot. Given the errors noted above, these figures cannot be considered to reliably represent the data collected. Although replicates are available for the figures, the authors are retracting this publication, due to the possibility of misrepresentation of the data. All of the authors believe that it is necessary to confirm that the conclusions drawn are corrected for publication in the future.

All of the authors have agreed to this retraction. The authors apologize to the readers and editors of *PLOS Genetics* for these errors.
